# Prognostic value of machine learning for brain computed tomography as a predictor of neurologic outcomes after cardiac arrest: a systematic review and meta-analysis

**DOI:** 10.1186/s13049-026-01565-w

**Published:** 2026-01-30

**Authors:** Kyung Hun Yoo, Juncheol Lee, Wonhee Kim, Bitnarae Kim, Elleah Jueun Chin, Jae-Guk Kim, Hyun-Young Choi, Jaehoon Oh

**Affiliations:** 1 Department of Emergency Medicine, Hanyang University College of Medicine, Seoul, Republic of Korea; 2 Department of Emergency Medicine, Kangnam Sacred Heart Hospital, Hallym University College of Medicine, 1 Singil-Ro, Yeongdeungpo-Gu, Seoul, 07441 Republic of Korea; 3 Hallym Biomedical Informatics Convergence Research Center, Kangnam Sacred Heart Hospital, Hallym University College of Medicine, Seoul, Republic of Korea; 4 Department of Digital Health, Samsung Advanced Institute for Health Science & Technology (SAIHST), Sungkyunkwan University, Seoul, Republic of Korea

**Keywords:** Heart arrest, Machine learning, Neuroimaging, Prognosis, Computed tomography, Radiography, Systematic review

## Abstract

**Background:**

The gray-to-white matter ratio (GWR) on brain computed tomography (CT) is used to predict neurological outcomes after cardiac arrest. Even though automated methods, such as automatic GWR and machine learning, have been compared to manual GWR, the superiority remains unknown. Therefore, we conducted a systematic review and meta-analysis to compare the diagnostic accuracy of these three CT-based methods.

**Methods:**

We systematically searched MEDLINE, EMBASE, Web of Science, Scopus, and IEEE Xplore and included studies evaluating neurological outcomes using the Cerebral Performance Category Scale. We performed a subgroup analysis to compare machine learning with manual or automatic GWR measurements. The Prediction model Risk of Bias ASsessment Tool was used to assess the risk of bias and applicability. Diagnostic accuracy for predicting poor neurological outcomes was evaluated using the pooled diagnostic odds ratio (DOR) and pooled area under the curve (AUC).

**Results:**

In total, 1594 patients from seven observational studies were included. Machine learning showed significantly higher diagnostic accuracy (pooled AUC, 0.813; pooled DOR, 14.02; 95% confidence interval [CI], 6.51–30.18; *I*^*2*^ = 63.1%) than manual GWR measurement (pooled AUC, 0.755; pooled DOR, 5.16; 95% CI, 3.75–7.08, *I*^*2*^ = 0%; *p* = 0.02). Machine learning showed statistically equivalent diagnostic accuracy, although it was numerically lower than automatic GWR measurement (pooled AUC, 0.832; pooled DOR, 11.92, 95% CI, 7.55–18.82; *I*^*2*^ = 24.3%; *p* = 0.72) for predicting poor neurological outcomes.

**Conclusion:**

Machine learning in brain CT may have significant diagnostic value for predicting poor neurological outcomes in post-cardiac arrest patients. Machine learning may be comparable to automatic GWR measurement and outperform manual GWR measurement in terms of diagnostic accuracy.

**Supplementary Information:**

The online version contains supplementary material available at 10.1186/s13049-026-01565-w.

## Introduction

Post-cardiac arrest syndrome is a unique and complex combination of pathophysiological processes, including post-cardiac arrest brain injury [1, 2]. Brain injury, also known as hypoxic-ischemic brain injury (HIBI), is a major cause of mortality and neurological impairment [3]. Neuroimaging can be useful for identifying structural brain injury following cardiac arrest, with computed tomography (CT) being the most commonly used modality [4]. On brain CT, brain edema can be measured using the gray-white matter ratio (GWR), which is defined as the proportion of the density of gray matter to white matter [4, 5].

2025 International Liaison Committee on Resuscitation Consensus on Science With Treatment Recommendations suggest using GWR on CT for predicting neurological outcome [6, 7]. However, a precise definition for imaging findings is not provided; instead, they recommend that CT evidence of "reduced GWR" be used to predict poor neurological outcomes [8, 9]. In manual GWR (mGWR) measurement, the GWR at the basal ganglia level has the highest diagnostic accuracy for neuroprognosis [5]. Nevertheless, mGWR measurement is a time-consuming method, and its accuracy can be affected by some measurement factors. In terms of the time point of brain CT scan, the accuracy of mGWR measurement on early CT scans can differ from that on delayed CT scans after return of spontaneous circulation (ROSC). The measurement level of the CT scan or the experience of the clinician are also important factors affecting the accuracy of mGWR measurement [10]. To overcome these limitations, automatic GWR (aGWR) measurement was developed, which seems to be more objective and consistent than mGWR measurement [11–14]. Recently, some investigators have begun to use machine learning (ML) models on brain CT scans [15–17]. This is because ML has the strength to handle more complex and high-dimensional data [18]. However, we still do not know whether aGWR measurement or ML is more accurate than mGWR measurement for predicting neurological outcomes.

Therefore, we performed a systematic review and meta-analysis to compare mGWR measurement, aGWR measurement, and ML. This meta-analysis aimed to compare and evaluate the accuracy of these diagnostic tests on brain CT scans for predicting neurological outcomes in post-cardiac arrest patients.

## Methods

This systematic review and meta-analysis were performed according to the Prediction model Risk Of Bias ASsessment Tool (PROBAST) and the CHecklist for critical Appraisal and data extraction for systematic Reviews of prediction Modelling Studies (CHARMS) [19, 20]. The study protocol was approved by PROSPERO during the initial stages of the study (registration number: CRD42024611026).

### Search strategy

An extensive database search was performed using MEDLINE, EMBASE, Web of Science, Scopus, and IEEE Xplore for all studies published in English between January 1, 1970, and December 17, 2025. The following index terms and related terms were used in the search: ‘Algorithms,’ ‘Artificial Intelligence,’ ‘Decision Support System,’ ‘Heart Arrest,’ ‘Ventricular Fibrillation,’ ‘Cardiopulmonary Resuscitation,’ and ‘Computed Tomography.’ The full search strategy is presented in Supplementary Table 1.

### Study selection

According to pre-defined study selection criteria, screening of the studies was performed independently by two reviewers (W.K. and K.H.Y.). Two reviewers checked the titles and abstracts for relevance, and a full-text review was subsequently performed. The inclusion criteria were as follows: (1) peer-reviewed studies in English; (2) studies with neurological outcome prediction of cardiac arrest patients from brain CT scans; (3) studies with clear information on prediction methods and neurological outcomes; and (4) studies with clear information on the dataset and data source. The exclusion criteria were as follows: (1) non-original articles, (2) unpublished papers or abstract-only publications, (3) publications in non-English languages, (4) reviews, (5) non-human or animal studies, (6) studies including participants under 18 years of age, and (7) case reports and case series.

### Data extraction

Two reviewers (B.K. and E.J.C.) performed data extraction using a standardized data collection instrument. The following variables were extracted: author information, publication year and title, publication journal, type of ML model used, data source, data collection period, sample size, study setting, inclusion and exclusion criteria, age of participants, outcome definition, variable types used for prediction, outcome to be predicted (good/poor neurological outcome), and model performance results (area under the curve [AUC], sensitivity, specificity, accuracy, negative predictive value [NPV], and positive predictive value [PPV]).

For the extracted ML models, the supervised ML models developed for classification techniques were of interest. The extracted ML models were Logistic Regression (LR), Convolutional Neural Networks (CNNs), Random Forest (RF), and Support Vector Machine (SVM).

A 2 × 2 classification table was generated for the ML models used in each study. The 2 × 2 tables are presented as follows: true positive (TP) (good neurological outcome), false positive (FP) (poor neurological outcome but falsely predicted as good), true negative (TN) (poor neurological outcome), and false negative (FN) (good neurological outcome but falsely predicted as poor). The TP, FP, TN, and FN values were extracted from the model’s sensitivity, specificity, accuracy, NPV, PPV, and AUC results and calculated as follows:

- *Sensitivity* = *TP/(TP* + *FN)*
- *Specificity* = *TN/(TN* + *FP)*
- *Accuracy* = *(TP* + *TN)/(TP* + *FP* + *FN* + *TN)*
- *PPV* = *TP/(TP* + *FP)*
- *NPV* = *TN/(FN* + *TN)*

For studies not presenting the result outcomes as numerical metrics but only showing the AUC plot, Youden’s index (Youden’s *J* statistic), defined as *J* = *sensitivity* + *specificity – 1,* was used to obtain TP, FP, TN, and FN values. The maximum *J*-value of the AUC plot was used to select the optimal threshold value that best represented the diagnostic test results [21].

### Quality assessment

Quality assessment was independently performed by two reviewers (J.L. and K.H.Y.) using the CHARMS checklist and PROBAST to assess the risk of bias (ROB) and concerns regarding applicability. The quality of each study was evaluated as one of three categories: ‘Low ROB,’ ‘High ROB,’ or ‘Unclear.’ The domains included patient selection, predictor assessment, ROB introduced by the outcome, applicability concerns, and ROB introduced by the analysis. Any disagreements between the two reviewers were resolved by a third reviewer (W.K.).

### GWR measurement

**Fig. 1:**
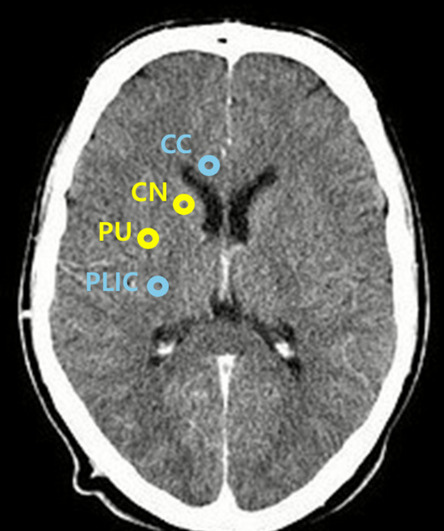
The GWR measurement at the basal ganglia level using brain CT. The calculation methods are as follows: BG-GWR = (CN + PU)/(CC + PLIC); simplified BG-GWR = PU/PLIC. Abbreviations: CT, computed tomography; GWR, gray-to-white matter ratio; BG-GWR, GWR measured at the basal ganglia level; CC, corpus callosum; CN, caudate nucleus; PU, putamen; PLIC, posterior limb of the internal capsule

In the included studies, the GWR was measured using either previously reported manual measurements, automatically computed methods, or ML models. As shown in Fig. 1, the GWR values at the basal ganglia level (BG-GWR) were measured bilaterally for the caudate nucleus (CN), putamen (PU), corpus callosum (CC), and posterior limb of the internal capsule (PLIC).The GWR values at the basal ganglia level (BG-GWR) were recorded bilaterally for the caudate nucleus (CN), putamen (PU), corpus callosum (CC), posterior limb of the internal capsule (PLIC).

The mGWR was calculated using one of the previously reported methods as follows:

1. *Simplified BG-GWR* = *PU/PLIC*
2. *BG-GWR* = *(CN* + *PU)/(CC* + *PLIC)*

The aGWR measurement is a method in which the GWR is computed using image-processing techniques of image registration and segmentation using brain CT images. The mean Hounsfield units (HUs) in the regions of interest (ROIs) are automatically measured, and the GWR is calculated using one of the following methods:

1. *Automated (simplified) BG-GWR* = *PU/PLIC*
2. *Automated BG-GWR* = *(CN* + *PU)/(CC* + *PLIC)*
3. *Automated GWR* = *Gray Matter/White Matter*

In studies that used ML methods, the models used were Very Deep Convolutional Networks for Large-Scale Image Recognition (VGG19 CNN) with transfer learning, LR, Linear SVM, Radial Basis Function kernel SVM, RF, and Elastic net regularized LR.

Nakamoto et al., 2024 utilized four distinct algorithms (LR, Linear SVM, Radial Basis Function SVM, RF) to analyze over 1,100 high-dimensional quantitative imaging biomarkers. To ensure model robustness and prevent overfitting, the authors implemented dimensionality reduction via LASSO and SVM Recursive Feature Elimination, alongside hyperparameter tuning through an AUC-based grid search with threefold cross-validation. Gramespacher et al., 2024 used a supervised classifier based on an elastic net regularized logistic regression model, which combined L1 and L2 penalties to improve prediction accuracy while handling the high correlation between 166 brain regions. The model was trained using stratified tenfold cross-validation on a development set and validated against an independent hold-out sample to ensure generalizability. Kawai et al., 2023 implemented a deep learning approach using the VGG19 CNN architecture with transfer learning from a pre-trained dataset. The model used raw head CT images and incorporated data augmentation and early stopping during training to minimize overfitting.

### Statistical analysis

The main analysis was an investigation of the neurological outcome of post-cardiac arrest patients based on the GWR on brain CT images. A meta-analysis of the evaluation metrics of AUC, sensitivity, and specificity of the included models was conducted, and a subgroup analysis was performed for the mGWR measurement, aGWR measurement, and ML methods to compare the differences in the diagnostic performance of each approach used in the included studies.

The main and subgroup analysis were performed using pooled diagnostic odds ratio (DOR), pooled sensitivity, and pooled specificity analyses using TP, TN, FP, and FN values. A Summary Receiver Operating Characteristic (SROC) curve was used to summarize the overall performance of the diagnostic tests. For the main and subgroup analysis, R Studio version 4.2.1 (R: A Language and Environment for Statistical Computing, R Core Team, R Foundation for Statistical Computing, Vienna, Austria, 2025, http://www.R-project.org/) was used for the statistical analysis, and *p*-value ≥ 0.05 was considered not significant for each compared subgroup. The ‘meta’ package in R was used for sensitivity, specificity, and DOR analysis, and the ‘mada’ package was used for the SROC curve analysis. Heterogeneity was estimated using the *I*^*2*^ statistic, which represents the percentage of the total inconsistency due to the true differences between the studies rather than variation resulting from chance, and values of 25%, 50%, and 75% were considered to indicate low, moderate, and high heterogeneity.

We have provided the PRISMA checklist as Supplementary Table 2.

## Results

### Study selection

**Fig. 2:**
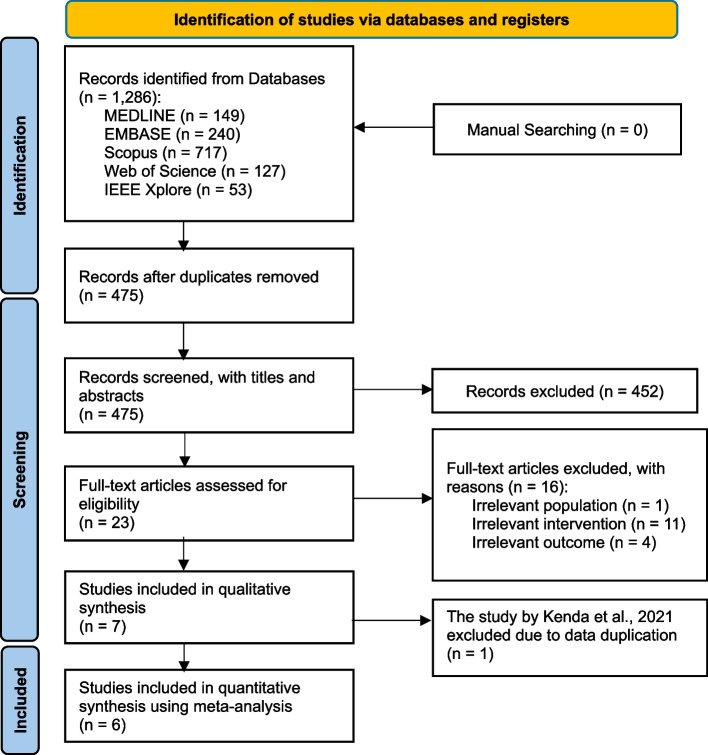
Flow chart

A flow diagram of the systematic review is shown in Fig. 2. A total of 1,286 studies were identified through literature search. After scanning the titles and abstracts, we discarded 811 duplicate articles retrieved through multiple search engines. An additional 452 irrelevant articles were excluded based on the titles and abstracts. After reviewing the full texts of the 23 remaining articles, 16 irrelevant articles were excluded as Supplementary Table 3. Finally, seven observational studies were included in this systematic review [11–17].

### Study characteristics and quality assessment

The included studies were published between 2016 and 2024, and the enrollment periods ranged from 2005 to 2021. A total of 1594 patients were included in the meta-analysis. The diagnostic accuracies of ML, aGWR measurement, and mGWR measurement were evaluated in three, four, and four studies, respectively. The mean time interval from cardiac arrest to the brain CT scans was within 24 h in five studies and more than 24 h in two studies (Kenda et al., 2021 [11] and Gramespacher et al., 2024 [16]). In five studies, poor neurological outcomes were defined as Cerebral Performance Category (CPC) grades 3, 4, and 5, while two studies (Kenda et al., 2021 [11] and Kenda et al., 2022 [12]) defined CPC grades 4 and 5 as poor neurological outcomes. Characteristics of the studies included in this meta-analysis are summarized in Table 1, and the diagnostic modalities are shown in Table 2.

The quality of the included studies was assessed using PROBAST (Supplementary Fig. 1). In the summary of ROB assessment, low ROB was 71% and high ROB was 29%, originating from the analysis domain of two studies (Hanning et al., 2016 [14] and Kenda et al., 2022 [12]). In the summary of applicability assessment, 86% low concern was shown, and 14% was unclear in the predictor domain of one study (Kenda et al., 2021 [11]). Through a systematic review, we found that the study by Kenda et al. in 2021 [11] contained duplicate data from the study by Kenda et al. in 2022 [12], as evidenced by author’s name, hospital’s name, and recruitment period in Table 1. Consequently, the study by Kenda et al. in 2021 [11] was excluded from the meta-analysis.

### Deviations from the protocol and post-hoc decisions

When conducting meta-analysis, overlapping cohorts are not permitted under the PROSPERO protocol. Following a systematic review, we noticed that the study by Kenda et al. in 2021 [11] contained duplicate data from the study by Kenda et al. in 2022 [12], as evidenced by the author's name, hospital name, and recruitment period listed in Table 1.

As a result, despite being included in the systematic review of Table 1, Kenda 2021 [11] was finally excluded from the meta-analysis by post-hoc decision, as shown in the PRISMA flowchart of Fig. 2.

### Comparison of diagnostic accuracy for poor neurological outcomes between ML, aGWR measurement, and mGWR measurement

We conducted a meta-analysis for all 6 included studies after removing the study by Kenda et al. in 2021 [11]. Forest plots of the sensitivity and specificity of the ML, aGWR, and mGWR measurements on brain CT for poor neurological outcomes are shown in Supplementary Figs. 2 and 3. We found no significant differences in sensitivity and specificity between these modalities (*p*-value for sensitivity = 0.7349, *p*-value for specificity = 0.1065).

**Fig. 3:**
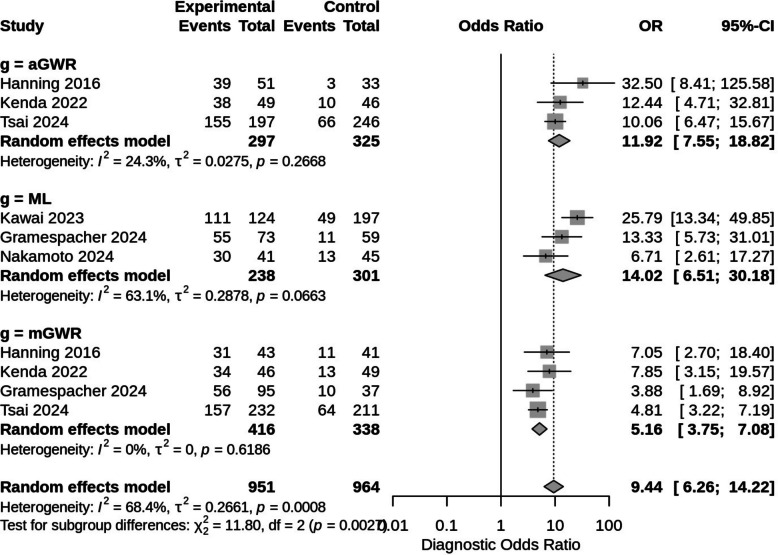
Forest plot of pooled diagnostic odds ratios for neurologic outcomes. The test for subgroup differences was significant between groups (*p*-value = 0.0027). # DOR subgroup 1 = ML vs mGWR (*p*-value = 0.0182). # DOR subgroup 2 = ML vs aGWR (*p*-value = 0.7214). # DOR subgroup 3 = aGWR vs mGWR (*p*-value = 0.0032). Abbreviations: DOR, diagnostic odds ratio; ML, machine learning; aGWR, automatic measurement of gray-to-white matter ratio; mGWR, manual measurement of gray-to-white matter ratio

**Fig. 4:**
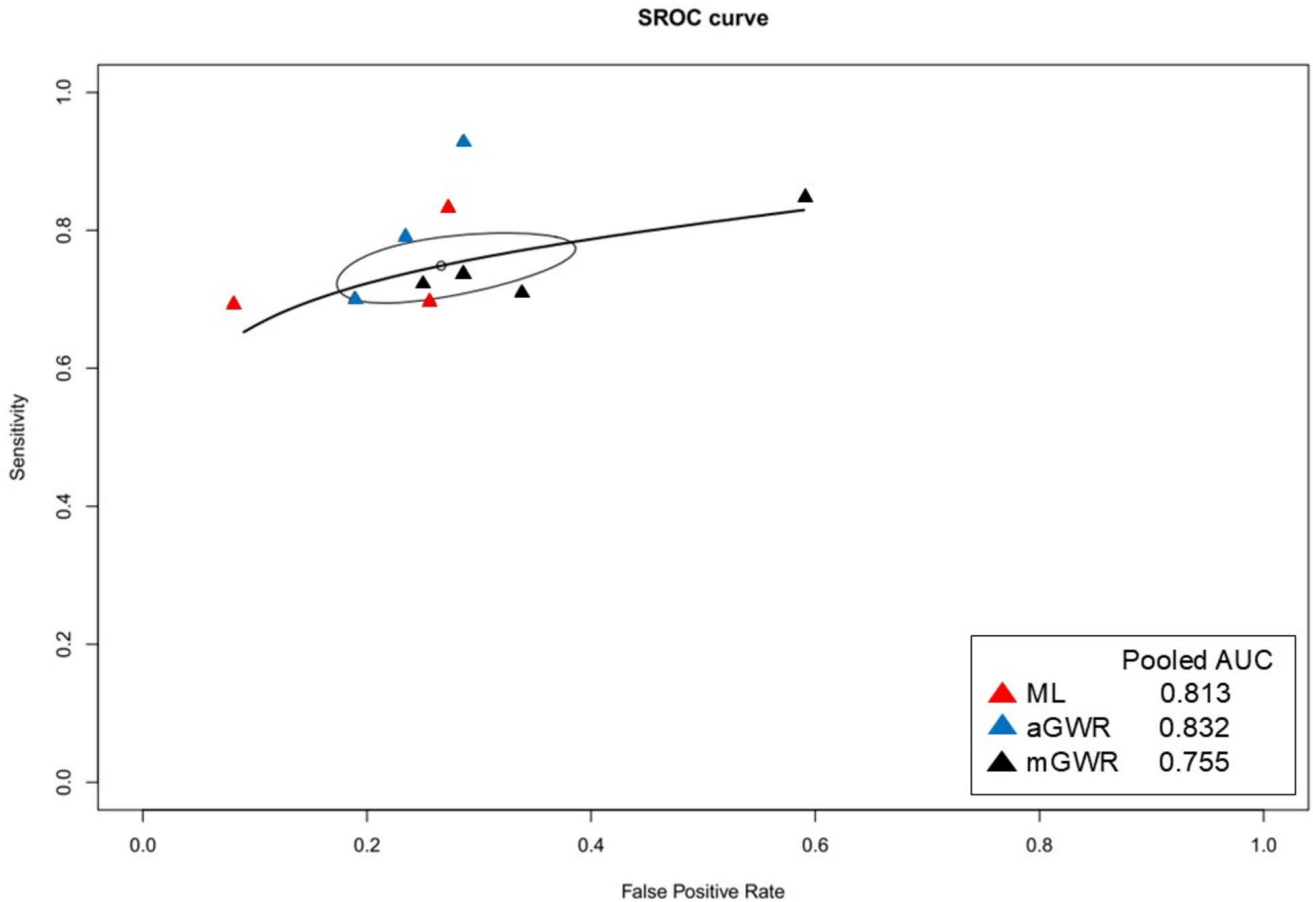
Summary receiver operating characteristic (SROC) curve for neurologic outcomes. Abbreviations: SROC, summary receiver operating characteristic; AUC, area under the curve; ML, machine learning; aGWR, automatic measurement of gray-to-white matter ratio; mGWR, manual measurement of gray-to-white matter ratio. # ML pooled AUC 0.813, *I*^*2*^ = 0%. # aGWR pooled AUC 0.832, *I*^*2*^ = 0%. # mGWR pooled AUC 0.755, *I*^*2*^ = 0%

The meta-analysis also compared the diagnostic accuracy of ML, aGWR, and mGWR measurements on brain CT using the DOR and the pooled AUC using the summary receiver operating characteristic (SROC) curve (Figs. 3 and 4). In the comparison between ML and mGWR measurements, ML showed significantly higher diagnostic accuracy (pooled AUC, 0.813; *I*^*2*^ = 0%; pooled DOR, 14.02; 95% confidence interval [CI], 6.51–30.18; *I*^*2*^ = 63.1%) than mGWR measurement (pooled AUC, 0.755; *I*^*2*^ = 0%; pooled DOR, 5.16; 95% CI 3.75–7.08; *I*^*2*^ = 0%; *p* = 0.0182). In the comparison between ML and aGWR measurements, ML showed equal diagnostic accuracy to aGWR measurement (pooled AUC, 0.832; *I*^*2*^ = 0%; pooled DOR, 11.92; 95% CI, 7.55–18.82; *I*^*2*^ = 24.3%; *p* = 0.7214) for the prediction of poor neurological outcomes. In the comparison between aGWR and mGWR measurements, aGWR measurement also showed significantly higher diagnostic accuracy than mGWR measurement (*p* = 0.0032).

We also performed a meta-analysis of brain CT scan timing before and after the 24-h mark from hospital arrival to determine the effect of brain CT timing on diagnostic accuracy for ML, aGWR measurement, and mGWR measurement. The early brain CT group included CTs performed within 24 h of hospital arrival. The second group was designated the late brain CT group. In Supplementary Fig. 4, the early brain CT group had a lower pooled sensitivity than the late brain CT group (early CT group: 0.72; 95% CI, 0.68–0.75; *I*^*2*^ = 27.6%; late CT group: 0.84; 95% CI, 0.77–0.89; *I*^*2*^ = 0%; *p* = 0.0032). However, the pooled specificity between the early and late brain CT groups was not significantly different (early CT group: 0.77; 95% CI, 0.7–0.83; late CT group: 0.57; 95% CI, 0.27–0.84; *p* = 0.1924) and showed high heterogeneity, as shown in Supplementary Fig. 5.

In addition, there was no significant difference in the pooled DOR between the early and late brain CT groups (early CT group: 10.09; 95% CI, 6.38–15.96; late CT group: 7.18; 95% CI, 2.14–24.07; *p* = 0.6054) and high heterogeneity was shown for both groups in Supplementary Fig. 6. In each subgroup DOR analysis for ML and mGWR measurement, there was no significant difference in the pooled DOR between the early and late brain CT groups in either diagnostic modality (ML: *p* = 0.9689; mGWR: *p* = 0.4681; aGWR: *p*-value was unavailable because of the absence of a late brain CT group comparison).

### Sensitivity analysis

We also performed sensitivity analysis to address heterogeneity issues. All subgroups with significant heterogeneity were identified.

First, we assessed the impact of data duplication by Kenda 2021 [11]. The pooled DOR with Kenda 2021 [11] demonstrated high accuracy despite significant heterogeneity (pooled DOR: 24.55; 95% CI 6.84–88.08; I^2^ = 63.1%). After excluding Kenda 2021 [11], the pooled DOR's heterogeneity was significantly reduced, despite a lower accuracy (pooled DOR: 11.92; 95% CI: 7.55–18.82; I^2^ = 24.3%). Figure 3's forest plot revealed significant heterogeneity in the pooled DOR of ML subgroups (subgroup I^2^: ML 63.1%; aGWR 24.3%; mGWR 0%). Following sensitivity analysis for pooled DOR, excluding Kawai 2023 [17] from ML subgroups was the most effective method for significantly reducing heterogeneity (subgroup I^2^: ML 11.2%). Although excluding Nakamoto 2024 [15] for ML reduced heterogeneity (subgroup I^2^: ML 31.4%), it was less effective than excluding Kawai 2023 [17]. In this analysis, ML and aGWR still had higher pooled DOR than mGWR (*p* = 0.0078 in the test of subgroup differences).

In Supplementary Fig. 2, pooled sensitivity of ML and aGWR subgroups showed high heterogeneity (subgroup I^2^: ML 57.4%; aGWR 77%; mGWR 0%). Gramespacher 2024 [16] for ML and Hanning 2016 [14] for aGWR were excluded to reduce heterogeneity. Following sensitivity analysis, the heterogeneity of pooled sensitivity was significantly reduced (subgroup I^2^: ML 0%; aGWR 36.1%), with no significant difference between the subgroups.

In Supplementary Fig. 3, pooled specificity of ML and mGWR subgroups showed high heterogeneity (subgroup I^2^: ML 87%; mGWR 83.4%; aGWR 7.9%). Kawai 2023 [17] for ML and Gramespacher 2024 [16] for mGWR were excluded to reduce heterogeneity. Following sensitivity analysis, ML and mGWR heterogeneities were significantly reduced (subgroup I^2^: ML 0%, mGWR 0%). There is no significant difference among those subgroups.

In Supplementary Fig. 5, the pooled specificity of CT timing subgroups revealed high heterogeneity (subgroup I^2^: early CT (t < 24 h) 80.1%; late CT (t > 24 h) 92.4%). Following sensitivity analysis for pooled specificity, the heterogeneity of the early CT subgroup was reduced (subgroup I^2^: 53.5%), but not significantly, as expected. The late CT subgroup was excluded because only two studies were insufficient to conduct sensitivity analysis, despite the fact that there is still significant heterogeneity.

### Publication bias

We conducted Egger’s LR test to evaluate funnel plot asymmetry. This test demonstrated that no publication bias existed in the meta-analysis (*p* = 0.3217).

## Discussion

This systematic review and meta-analysis evaluated the effectiveness of three brain CT methods (mGWR measurement, aGWR measurement, and ML) in predicting neurological outcomes in post-cardiac arrest patients. The results indicate clear distinctions in the predictive performance of these methodologies. The meta-analysis results showed that ML significantly outperformed mGWR measurement in terms of diagnostic accuracy. Furthermore, aGWR measurement showed a higher pooled AUC and pooled DOR than ML, but these metrics alone were insufficient to conclude definitive superiority due to overlapping CIs and lack of statistical significance, indicating that both methods are comparably effective.

There was no statistically significant difference between ML and aGWR measurement, suggesting that ML was not inferior to aGWR in terms of diagnostic accuracy. ML models offer distinct advantages that make them promising tools for prognostication. Unlike aGWR measurement, which depends on predefined region-based measurements, ML algorithms can learn from high-dimensional image patterns and incorporate multimodal data, such as clinical variables, imaging texture features, and time-series trends, into outcome prediction.

Gramespacher et al. emphasized that integrating clinical and radiologic features using elastic net regularization enhanced early prognostic performance [16], while Kawai et al. demonstrated that ML-based models using early CT scans could capture subtle injury patterns that are not visible to conventional metrics [17].

Nevertheless, the modest sample sizes of the studies limited the ability of the ML models to demonstrate significant superiority over aGWR measurement methods, particularly deep learning methods such as CNNs, which remain dominant in image-based prediction tasks, as these approaches typically require large datasets to achieve their full potential [22]. The modest dataset size does not have the high-dimensional interactions that models such as CNN, LR, SVM, and Elastic Net Regression methods require and will increase the risk of overfitting [23]. Another point to consider is that all studies were performed only with a single-center dataset, which also presents challenges for improving model performance and hinders the model’s generalizability to other data [24]. On top of that, only three (Kenda et al. [12], Kawai et al. [17], and Nakamoto et al. [15]) of the included studies reported hyperparameter tuning [12, 15, 17]. Without adequate hyperparameter tuning, the performance of ML algorithms would have been constrained relative to what could have been achieved through more rigorous training strategies [25]. With a more suitable data size and training method, the adaptability of ML allows for ongoing model improvement as more annotated data becomes available, supporting real-time and individualized predictions [26]. Therefore, even if ML currently performs comparably to aGWR measurement, its scalability and flexibility highlight its future clinical value, particularly in settings requiring early, automated, and reproducible prognostic tools.

In addition, broader evidence supports the growing significance of ML in the early prognosis across neurological conditions [10]. A recent systematic review highlighted how ML models that combine imaging, clinical, biomarker, and electrophysiological data achieve strong predictive performance, suggesting that they can process multimodal information more comprehensively than traditional metrics [27].

One notable study applied an interpretable ML pipeline to post-cardiac arrest brain CT scans and demonstrated that gray matter texture analysis could yield accurate prognostic information within hours of ROSC, further cementing the potential role of ML in early decision-making.

These findings underscore the ability of ML approaches to extract subtle imaging patterns in stroke or post-arrest brain injuries, which are often imperceptible to human observers.

Regarding the timing of brain CT scans, this meta-analysis revealed no significant difference in the diagnostic accuracy of brain CT performed before or after the 24-h mark post-ROSC (*p* = 0.59). Beekman et al. suggested that early brain CT alone is insufficient for definitive prognostication and cautioned against the potential biases associated with its early use [10]. Additionally, Scheel et al. indicated that the sensitivity of GWR measurement markedly improved 6 h post cardiac arrest, thereby advocating for delayed imaging [28]. Conversely, Pereira et al. reported that early GWR measurements provide useful prognostic insights that closely align with our findings [29]. The findings of this study provide evidence against the necessity of delayed CT imaging, suggesting that early CT imaging within 24 h could be effective for prognostication. This result potentially challenges the established preference for delayed imaging and indicates that clinicians may reliably utilize early CT scans for prognosis evaluation without compromising accuracy.

Accurate prognostic evaluation within the first 24 h after cardiac arrest not only guides medical decision-making but also significantly impacts resource allocation. Our findings suggest that early assessment yields comparable prognostic value, thereby enabling timely clinical decisions. The early identification of patients with a favorable prognosis allows healthcare systems to reallocate critical staff time and intensive care resources more efficiently, focusing on those most likely to benefit. Several studies have suggested that faster prognostic evaluation in post-cardiac arrest patients may contribute to reduced medical costs and resource burdens [30, 31]. The early identification of patients who are unlikely to recover can potentially limit unnecessary prolongation of intensive care, whereas those with a favorable prognosis can receive appropriately aggressive management. Thus, early prognostication may support more efficient use of critical care resources without compromising the quality of care.

Early brain CT has already been used to rule out acute cerebral hemorrhage as the cause of arrest in PCAS patients [32]. However, automated analysis using ML or aGWR for early brain CT is not currently used to detect HIBI in emergency workflows. When integrating these automated analysis models, we must consider feasibility, cost-effectiveness, computational infrastructure requirements, and physician acceptance [33, 34]. In the long term, ML will be promising because its diagnostic accuracy will be dramatically improved if more big data is stacked. However, when considering short-term effects and cost-effectiveness, aGWR appears to be more feasible than ML. In future research, the diagnostic accuracy of ML and aGWR should be rigorously tested using prospective multicenter validation. Furthermore, CT-based models should be compared to other multimodal models that combine CT imaging, clinical data, and electrophysiology.

This study has some limitations. First, this meta-analysis relied on single-centered and retrospective observational design studies, which may have led to selection bias. Furthermore, small sample sizes, limited studies, and the lack of external validation may result in insufficient test power for the pooled estimate of the ML model. Second, when reconstructing TP, FP, TN, and FN using Youden's J statistic in small sample sizes, it can be mathematically unstable and lack robustness in obtaining the optimal threshold. Because heterogeneity among ML model types can be raised when treated as a single pooled group for meta-analysis, results may be distorted and unreliable. Third, the definition of poor neurological outcomes varied across the included studies; some studies classified CPC grades 3–5 as poor, whereas others defined CPC grades 4–5 as poor. This inconsistency introduces potential heterogeneity into the pooled diagnostic accuracy and may limit the comparability of the results. Finally, the PROBAST tool was used to evaluate the ROB. In the analysis domain, both the Hanning et al. and Kenda et al. studies were classified as having a high ROB. The following subdomains were mainly responsible for those high ROB issues: insufficient sample size, overfitting potential, unclear predictor handling, or missing data. Systemic distortion and an overestimation of pooled accuracy could result from these biases. As a result, we have to interpret these results cautiously, and additional reliable studies are still required.

## Conclusions

ML in brain CT may have significant diagnostic value for predicting poor neurological outcomes in post-cardiac arrest patients. ML may be comparable to aGWR measurement and outperform mGWR measurement in terms of diagnostic accuracy.

## Trial registration

PROSPERO registered ID: CRD42024611026.

## Supplementary Information

Supplementary Material 1: Supplementary Table 1. Search strategy.

Supplementary Material 2: Supplementary Table 2. PRISMA checklist.

Supplementary Material 3: Supplementary Table 3. Reason for exclusion from full-text review in flow chart.

Supplementary Material 4: Supplementary Fig. 1. Risk of bias and applicability assessment for included studies (A), summary of risk of bias assessment (B), and summary of applicability assessment (C).

Supplementary Material 5: Supplementary Fig. 2. Forest plot of pooled sensitivity for neurologic outcomes. Subgroup differences were not significant (*p* = 0.7349).

Supplementary Material 6: Supplementary Fig. 3. Forest plot of pooled specificity for neurologic outcomes. Subgroup differences were not significant (*p* = 0.1065).

Supplementary Material 7: Supplementary Fig. 4. Forest plot of the pooled sensitivity of brain computed tomography timing before and after the 24-h mark. Subgroup differences were significant between groups (*p* = 0.0032).

Supplementary Material 8: Supplementary Fig. 5. Forest plot of the pooled specificity of brain computed tomography timing before and after the 24-h mark. Subgroup differences were not significant (*p* = 0.1924).

Supplementary Material 9: Supplementary Fig. 6. Forest plot of the pooled diagnostic odds ratio of brain computed tomography timing before and after the 24-h mark. Subgroup differences were not significant (*p* = 0.6054). Abbreviations: ML, machine learning aGWR, automatic measurement of gray-to-white matter ratio; mGWR, manual measurement of gray-to-white matter ratio; CT, computed tomography; AUC, area under the curve. # Test before and after the 24-h mark in the ML group: *p* = 0.9689. # Test before and after the 24-h mark in the aGWR: Not available. # Test before and after the 24-h mark in the mGWR: *p* = 0.4681. # CT timing < 24 h: pooled AUC 0.734, *I*^*2*^ = 0%. # CT timing > 24 h: pooled AUC 0.835, *I*^*2*^ = 0%.

## Data Availability

The study protocol was registered in PROSPERO (CRD42024611026). All data extracted from included studies. Extracted data used for meta-analysis are available from the corresponding author upon reasonable request, while raw data from individual studies cannot be shared as they belong to the original authors.
