# Supplementary material for: Prognostic value of machine learning for brain computed tomography as a predictor of neurologic outcomes after cardiac arrest: a systematic review and meta-analysis
Source: Scand J Trauma Resusc Emerg Med. 2026 Jan 30;34:48. doi: 10.1186/s13049-026-01565-w (PMC12931003; doi:10.1186/s13049-026-01565-w)
Supplement: Supplementary file 1 — Supplementary Material 1: Supplementary Table 1. Search strategy. [file 13049_2026_1565_MOESM1_ESM.docx]

**Supplementary Table 1. Search strategy**

| **Database** | **Search term** |
| --- | --- |
| MEDLINE  #1: 1,392,282  #2: 122,150  #3: 944,577  **#4: 149** | 1. “Algorithms”[Mesh] OR "Artificial Intelligence"[Mesh] OR "Decision trees"[MeSH] OR "Decision Support Systems, Clinical"[Mesh] OR "Decision Making, Computer Assisted"[Mesh] OR "Decision Support Techniques"[Mesh] OR "Decision Support Systems, Clinical"[Mesh] OR “Diagnosis, Computer-Assisted”[MESH] OR machine learning[tiab] OR prediction algorithm*[tiab] OR prediction model*[tiab] OR neural network*[tiab] OR deep learning[tiab] OR artificial intelligence[tiab] OR AI[tiab] OR decision tree*[tiab] OR computational intelligence[tiab] OR machine intelligence[tiab] OR algorithm*[tiab] OR big data[tiab] OR bayesian[tiab] OR naïve bayes[tiab] OR k-nearest neighbour[tiab] OR decision support[tiab] OR random forest[tiab] OR support vector machine[tiab] OR SVM[tiab] OR Xgboost[tiab] OR adaboost[tiab] OR gradient boosting machine*[tiab] OR regression tree*[tiab] OR least squares[tiab] OR stepwise regression[tiab]  2. ("Out-of-Hospital Cardiac Arrest"[MeSH Terms] OR "Heart Arrest"[MeSH Terms] OR "Ventricular Fibrillation"[MeSH Terms] OR "Advanced Cardiac Life Support"[MeSH Terms] OR "Cardiopulmonary Resuscitation"[MeSH Terms] OR "Heart Massage"[MeSH Terms]) OR ("out of hospital cardiac arrest"[Title/Abstract] OR "out-of-hospital cardiac arrest"[Title/Abstract])  OR ("return of spontaneous circulation"[Title/Abstract] OR ROSC[Title/Abstract]) OR ("cardiac arrest"[Title/Abstract] OR "cardiovascular arrest"[Title/Abstract] OR "heart arrest"[Title/Abstract] OR "cardiopulmonary arrest"[Title/Abstract]) OR (asystole*[Title/Abstract]) OR ("pulseless electrical activity"[Title/Abstract]) OR ("advanced cardiac life support"[Title/Abstract] OR ACLS[Title/Abstract]) OR ("cardiopulmonary resuscitation"[Title/Abstract] OR CPR[Title/Abstract])  OR ("heart massage"[Title/Abstract])  3. "Tomography, X-Ray Computed"[Mesh] OR CT[tiab] OR "computed tomograph*"[tiab] OR "computer assisted tomograph*"[tiab]  4. #1 AND #2 AND #3 |
| EMBASE  #1: 772,186  #2: 307,417  #3: 1,689,901  **#4: 240** | 1. exp "artificial intelligence"/ or exp "computer prediction"/ or exp "decision tree"/ or exp "machine learning"/ or exp "artificial neural network"/ or "machine learning:ab,ti,kw".mp. or "prediction algorithm:ab,ti,kw".mp. or "prediction model:ab,ti,kw".mp. or "neural network:ab,ti,kw".mp. or "deep learning:ab,ti,kw".mp. or "artificial intelligence:ab,ti,kw".mp. or AI:ab,ti,kw.mp. or "decision tree:ab,ti,kw".mp. or "computational intelligence:ab,ti,kw".mp. or "machine intelligence:ab,ti,kw".mp. or algorithm:ab,ti,kw.mp. or "big data:ab,ti,kw".mp. or bayesian:ab,ti,kw.mp. or "naive bayes:ab,ti,kw".mp. or "k-nearest neighbour:ab,ti,kw".mp. or "decision support:ab,ti,kw".mp. or "random forest:ab,ti,kw".mp. or "support vector machine:ab,ti,kw".mp. or SVM:ab,ti,kw.mp. or Xgboost:ab,ti,kw.mp. or adaboost:ab,ti,kw.mp. or "gradient boosting machine:ab,ti,kw".mp. or "regression tree:ab,ti,kw".mp. or "least squares:ab,ti,kw".mp. or "stepwise regression:ab,ti,kw".mp. [mp=title, abstract, heading word, drug trade name, original title, device manufacturer, drug manufacturer, device trade name, keyword heading word, floating subheading word, candidate term word] 2. exp "out-of-hospital cardiac arrest"/ or ("out of hospital cardiac arrest" or "out-of-hospital cardiac arrest").ti,ab. or ("return of spontaneous circulation" or "ROSC").ti,ab. or exp "heart arrest"/ or "cardiac arrest".ti,ab. or "cardiovascular arrest".ti,ab. or "heart arrest".ti,ab. or asystole.ti,ab. or "pulseless electrical activity".ti,ab. or exp "ventricular fibrillation"/ or exp "advanced cardiac life support"/ or ("advanced cardiac life support" or ACLS).ti,ab. or exp "cardiopulmonary resuscitation"/ or CPR.mp. or "cardiopulmonary resuscitation".ti,ab. or exp "heart massage"/ 3. exp computer assisted tomography/ or "CT:ti,ab".mp. or "computed tomograph*:ti,ab".mp. or "computer assisted tomograph*:ti,ab".mp. [mp=title, abstract, heading word, drug trade name, original title, device manufacturer, drug manufacturer, device trade name, keyword heading word, floating subheading word, candidate term word] 4. #1 AND #2 AND #3 |
| IEEE Xplore  #1: 2,445,570  #2: 4,937  #3: 107,636  **#4: 53** | 1. ("Algorithms" OR "Artificial Intelligence" OR "Decision trees" OR "Decision Support Systems" OR "Decision Making" OR "Decision Support Techniques" OR "Diagnosis, Computer-Assisted" OR "machine learning" OR "prediction algorithm*" OR "prediction model*" OR "neural network*" OR "deep learning" OR "AI" OR "decision tree*" OR "computational intelligence" OR "machine intelligence" OR "algorithm*" OR "big data" OR "bayesian" OR "naïve bayes" OR "k-nearest neighbour" OR "decision support" OR "random forest" OR "support vector machine" OR "SVM" OR "Xgboost" OR "adaboost" OR "gradient boosting machine*" OR "regression tree*" OR "least squares" OR "stepwise regression")  2. ("Out-of-Hospital Cardiac Arrest" OR "Heart Arrest" OR "Ventricular Fibrillation" OR "Advanced Cardiac Life Support" OR "Cardiopulmonary Resuscitation" OR "Heart Massage" OR "return of spontaneous circulation" OR "ROSC" OR "cardiac arrest" OR "cardiovascular arrest" OR "cardiopulmonary arrest" OR "asystole*" OR "pulseless electrical activity" OR "ACLS" OR "CPR")  3. ("Tomography" OR "X-Ray Computed" OR "CT" OR "computed tomograph*" OR "computer assisted tomograph*")  4. #1 AND #2 AND #3 |
| Web of Science  #1: 2,587,592  #2: 54,888  #3: 469,015  **#4: 127** | 1. TS=("Algorithms" OR "Artificial Intelligence" OR "Decision trees" OR "Decision Support Systems" OR "Decision Making" OR "Decision Support Techniques" OR "Diagnosis, Computer-Assisted" OR "machine learning" OR "prediction algorithm*" OR "prediction model*" OR "neural network*" OR "deep learning" OR "AI" OR "decision tree*" OR "computational intelligence" OR "machine intelligence" OR "algorithm*" OR "big data" OR "bayesian" OR "naïve bayes" OR "k-nearest neighbour" OR "decision support" OR "random forest" OR "support vector machine" OR "SVM" OR "Xgboost" OR "adaboost" OR "gradient boosting machine*" OR "regression tree*" OR "least squares" OR "stepwise regression")  2. TS=("Out-of-Hospital Cardiac Arrest" OR "Heart Arrest" OR "Ventricular Fibrillation" OR "Advanced Cardiac Life Support" OR "Cardiopulmonary Resuscitation" OR "Heart Massage" OR "out of hospital cardiac arrest" OR "out-of-hospital cardiac arrest" OR "return of spontaneous circulation" OR "ROSC" OR "cardiac arrest" OR "cardiovascular arrest" OR "cardiopulmonary arrest" OR "asystole*" OR "pulseless electrical activity" OR "ACLS" OR "CPR")  3. TS=("Tomography, X-Ray Computed" OR "CT" OR "computed tomograph*" OR "computer assisted tomograph*")  4. #1 AND #2 AND #3 |
| Scopus  #1: 8,694,213  #2: 173,735  #3: 2,106,642  **#4: 717** | 1. TITLE-ABS-KEY("Algorithms" OR "Artificial Intelligence" OR "Decision trees" OR "Decision Support Systems" OR "Decision Making" OR "Decision Support Techniques" OR "Diagnosis, Computer-Assisted" OR "machine learning" OR "prediction algorithm*" OR "prediction model*" OR "neural network*" OR "deep learning" OR "AI" OR "decision tree*" OR "computational intelligence" OR "machine intelligence" OR "algorithm*" OR "big data" OR "bayesian" OR "naïve bayes" OR "k-nearest neighbour" OR "decision support" OR "random forest" OR "support vector machine" OR "SVM" OR "Xgboost" OR "adaboost" OR "gradient boosting machine*" OR "regression tree*" OR "least squares" OR "stepwise regression")  2. TITLE-ABS-KEY("Out-of-Hospital Cardiac Arrest" OR "Heart Arrest" OR "Ventricular Fibrillation" OR "Advanced Cardiac Life Support" OR "Cardiopulmonary Resuscitation" OR "Heart Massage" OR "return of spontaneous circulation" OR "ROSC" OR "cardiac arrest" OR "cardiovascular arrest" OR "cardiopulmonary arrest" OR "asystole*" OR "pulseless electrical activity" OR "ACLS" OR "CPR")  3. TITLE-ABS-KEY("Tomography" OR "X-Ray Computed" OR "CT" OR "computed tomograph*" OR "computer assisted tomograph*" OR "Computed Tomography")  4. #1 AND #2 AND #3 |
| All databases  **Total: 1,286** |  |
