# Supplementary material for: Prognostic value of machine learning for brain computed tomography as a predictor of neurologic outcomes after cardiac arrest: a systematic review and meta-analysis
Source: Scand J Trauma Resusc Emerg Med. 2026 Jan 30;34:48. doi: 10.1186/s13049-026-01565-w (PMC12931003; doi:10.1186/s13049-026-01565-w)
Supplement: Supplementary file 3 — Supplementary Material 3: Supplementary Table 3. Reason for exclusion from full-text review in flow chart. [file 13049_2026_1565_MOESM3_ESM.docx]

**Supplementary Table 3. Reason for exclusion from full-text review in flow chart**

|  | **Study ID** | **Reason for exclusion** | **Category of exclusion** |
| --- | --- | --- | --- |
| 1 | Radensky et al. 1997 | Not post-cardiac arrest patients | Irrelevant Population |
| 2 | Naples et al. 2009 | No reference of using machine learning or GWR on brain CT | Irrelevant Intervention |
| 3 | Chelly et al. 2012 | No reference of using machine learning or GWR on brain CT | Irrelevant Intervention |
| 4 | Gentsch et al. 2015 | No reference of using machine learning or aGWR on brain CT (mGWR only) | Irrelevant Intervention |
| 5 | Yamashita et al. 2016 | Not using CPC score to assess neurological outcome | Irrelevant Outcome |
| 6 | Geri et al.  2017 | No reference of using machine learning or GWR on brain CT | Irrelevant Intervention |
| 7 | Hannawi et al. 2020 | No reference of using machine learning or aGWR on brain CT (mGWR only) | Irrelevant Intervention |
| 8 | Moseby-Knappe et al. 2020 | No reference of using machine learning or GWR on brain CT | Irrelevant Intervention |
| 9 | Scarpino et al. 2021 | No reference of using machine learning or aGWR on brain CT (mGWR only) | Irrelevant Intervention |
| 10 | Elmer et al. 2022 | No reference of using machine learning or GWR on brain CT | Irrelevant Intervention |
| 11 | Elmer et al. 2022 | No reference of using machine learning or GWR on brain CT (EEG only) | Irrelevant Intervention |
| 12 | Mansour et al. 2022 | Not using CPC score to assess neurological outcome (HIBI only) | Irrelevant Outcome |
| 13 | Lee et al. 2023 | No reference of using machine learning or aGWR on brain CT (mGWR only) | Irrelevant Intervention |
| 14 | Liu et al. 2022 | Not using CPC score to assess neurological outcome (survival only) | Irrelevant Outcome |
| 15 | Molinski et al. 2024 | Not using CPC score to assess neurological outcome (HIBI only) | Irrelevant Outcome |
| 16 | Kawai et al. 2025 | No reference of using GWR on brain CT (machine learning only) | Irrelevant Intervention |

Abbreviations: GWR, gray-to-white matter ratio; aGWR, automatic GWR; mGWR, manual GWR;

CT, computed tomography; EEG, electroencephalography; HIBI, hypoxic-ischemic brain injury;

CPC, cerebral performance category
