# Supplementary material for: Prognostic value of machine learning for brain computed tomography as a predictor of neurologic outcomes after cardiac arrest: a systematic review and meta-analysis
Source: Scand J Trauma Resusc Emerg Med. 2026 Jan 30;34:48. doi: 10.1186/s13049-026-01565-w (PMC12931003; doi:10.1186/s13049-026-01565-w)
Supplement: Supplementary file 4 — Supplementary Material 4: Supplementary Fig. 1. Risk of bias and applicability assessment for included studies (A), summary of risk of bias assessment (B), and summary of applicability assessment (C). [file 13049_2026_1565_MOESM4_ESM.docx]

(A)


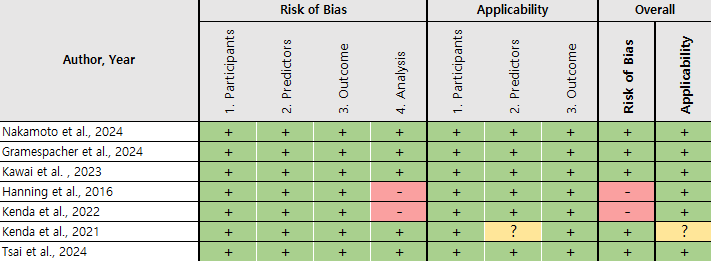


(B)

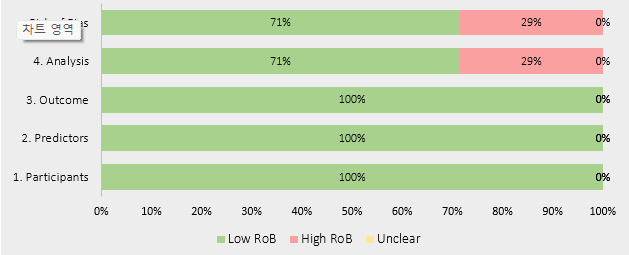


(C)

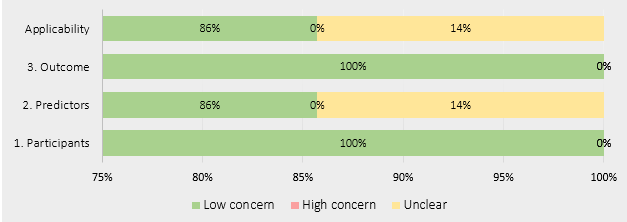

Supplementary Figure 1. Risk of bias and applicability assessment for included studies (A), summary of risk of bias assessment (B) and summary of applicability assessment (C)
