# Supplementary figures and images for: Prognostic value of machine learning for brain computed tomography as a predictor of neurologic outcomes after cardiac arrest: a systematic review and meta-analysis
Source: Scand J Trauma Resusc Emerg Med. 2026 Jan 30;34:48. doi: 10.1186/s13049-026-01565-w (PMC12931003; doi:10.1186/s13049-026-01565-w)

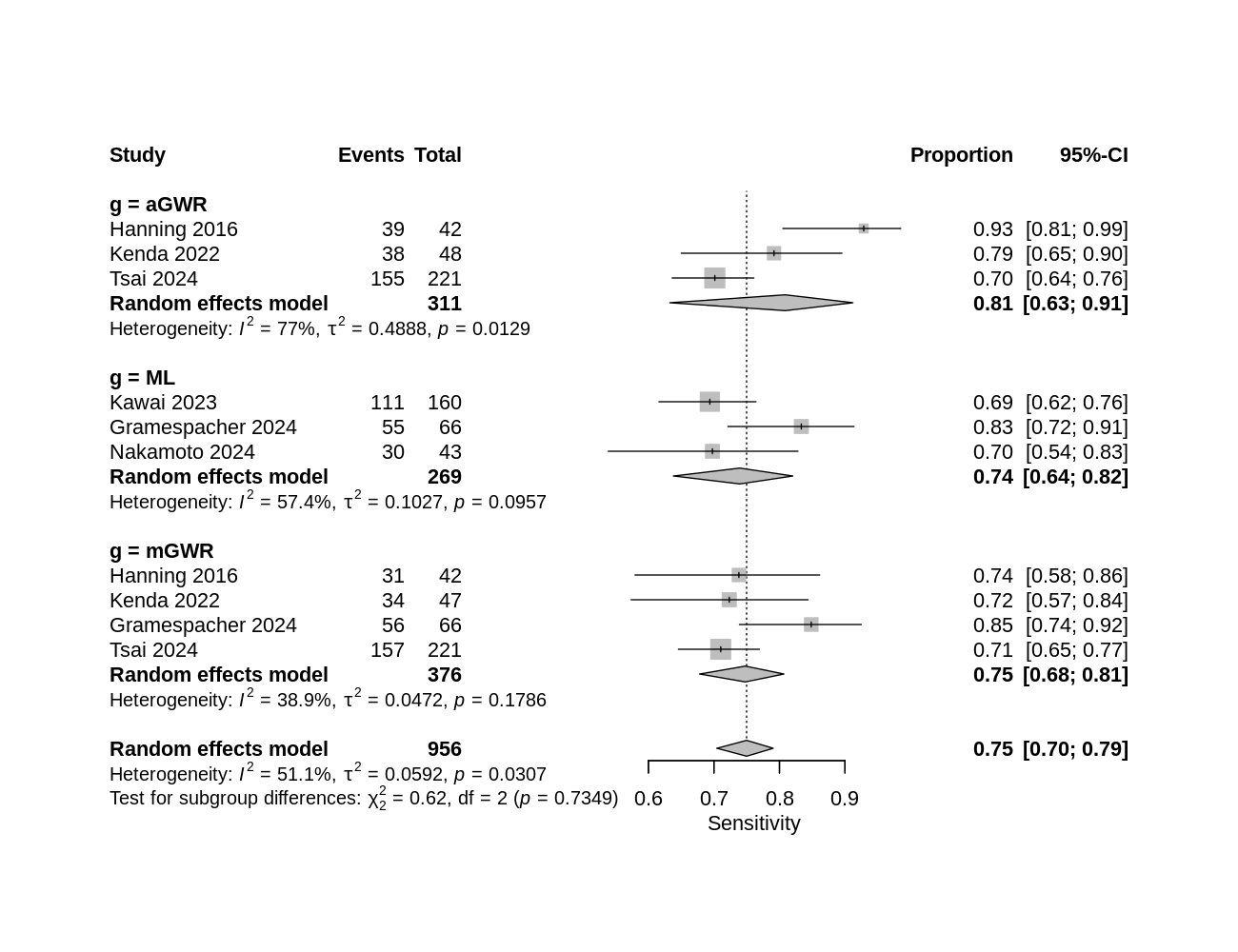

Supplement: Supplementary file 5 — Supplementary Material 5: Supplementary Fig. 2. Forest plot of pooled sensitivity for neurologic outcomes. Subgroup differences were not significant (p = 0.7349). [file 13049_2026_1565_MOESM5_ESM.tiff]

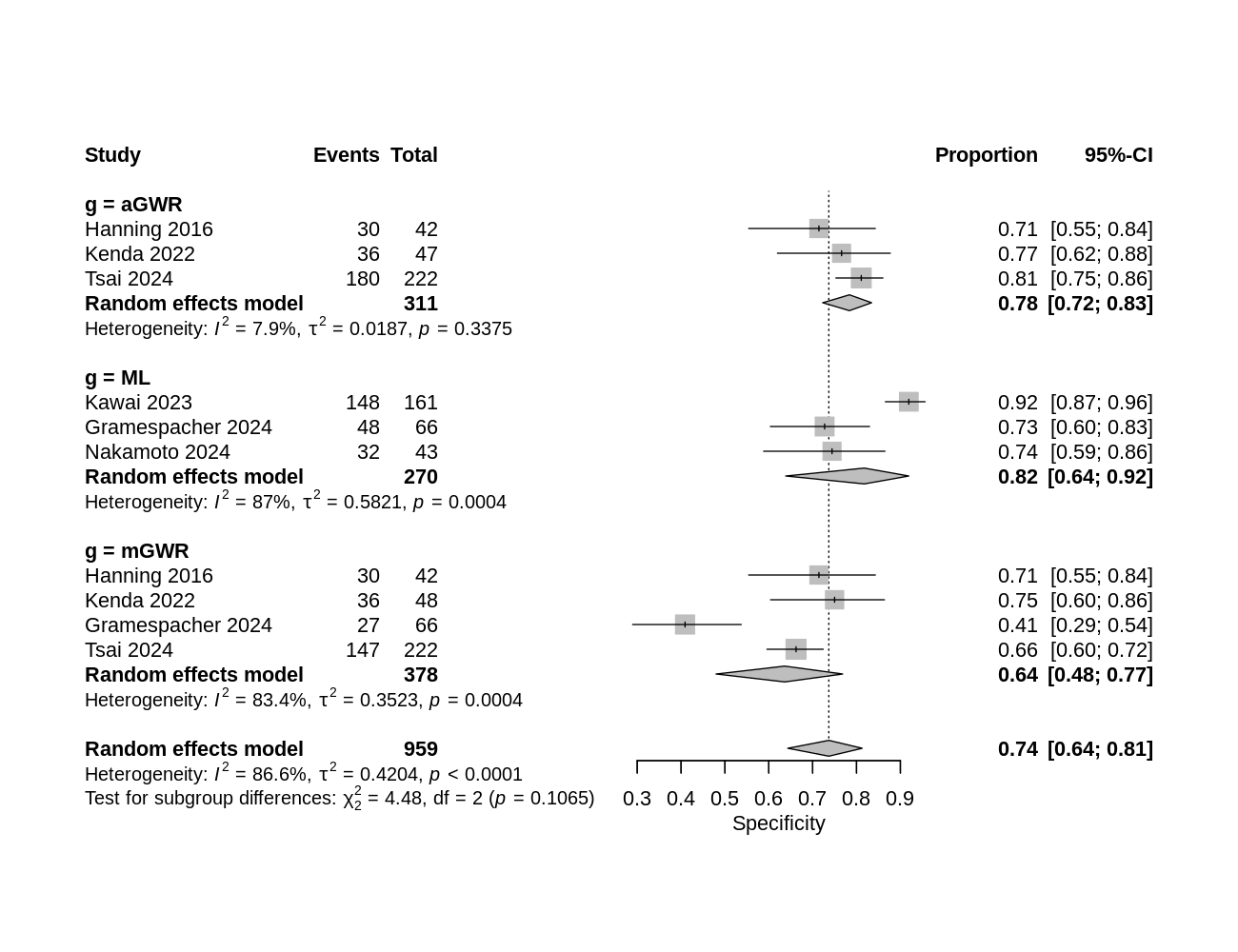

Supplement: Supplementary file 6 — Supplementary Material 6: Supplementary Fig. 3. Forest plot of pooled specificity for neurologic outcomes. Subgroup differences were not significant (p = 0.1065). [file 13049_2026_1565_MOESM6_ESM.tiff]

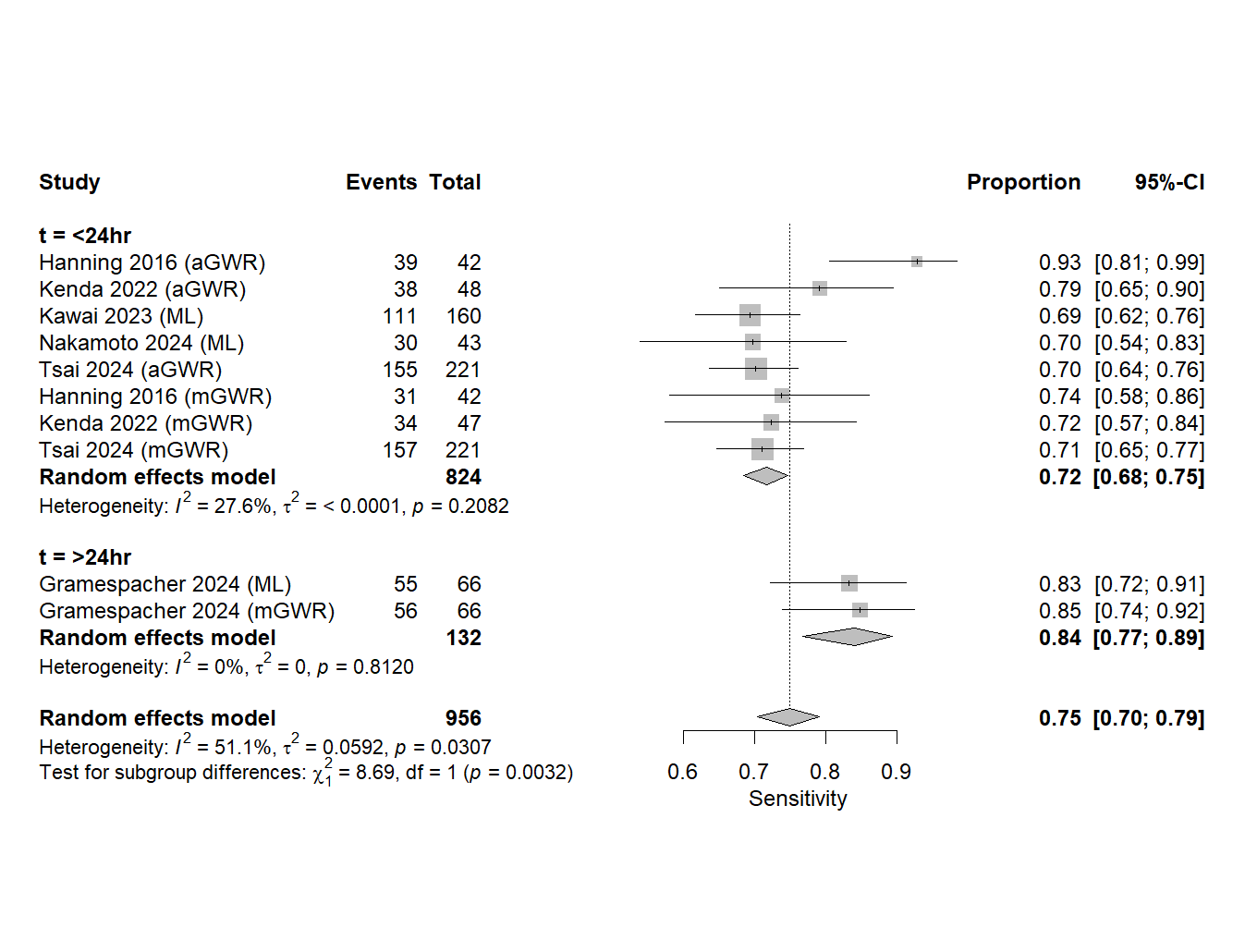

Supplement: Supplementary file 7 — Supplementary Material 7: Supplementary Fig. 4. Forest plot of the pooled sensitivity of brain computed tomography timing before and after the 24-h mark. Subgroup differences were significant between groups (p = 0.0032). [file 13049_2026_1565_MOESM7_ESM.tiff]

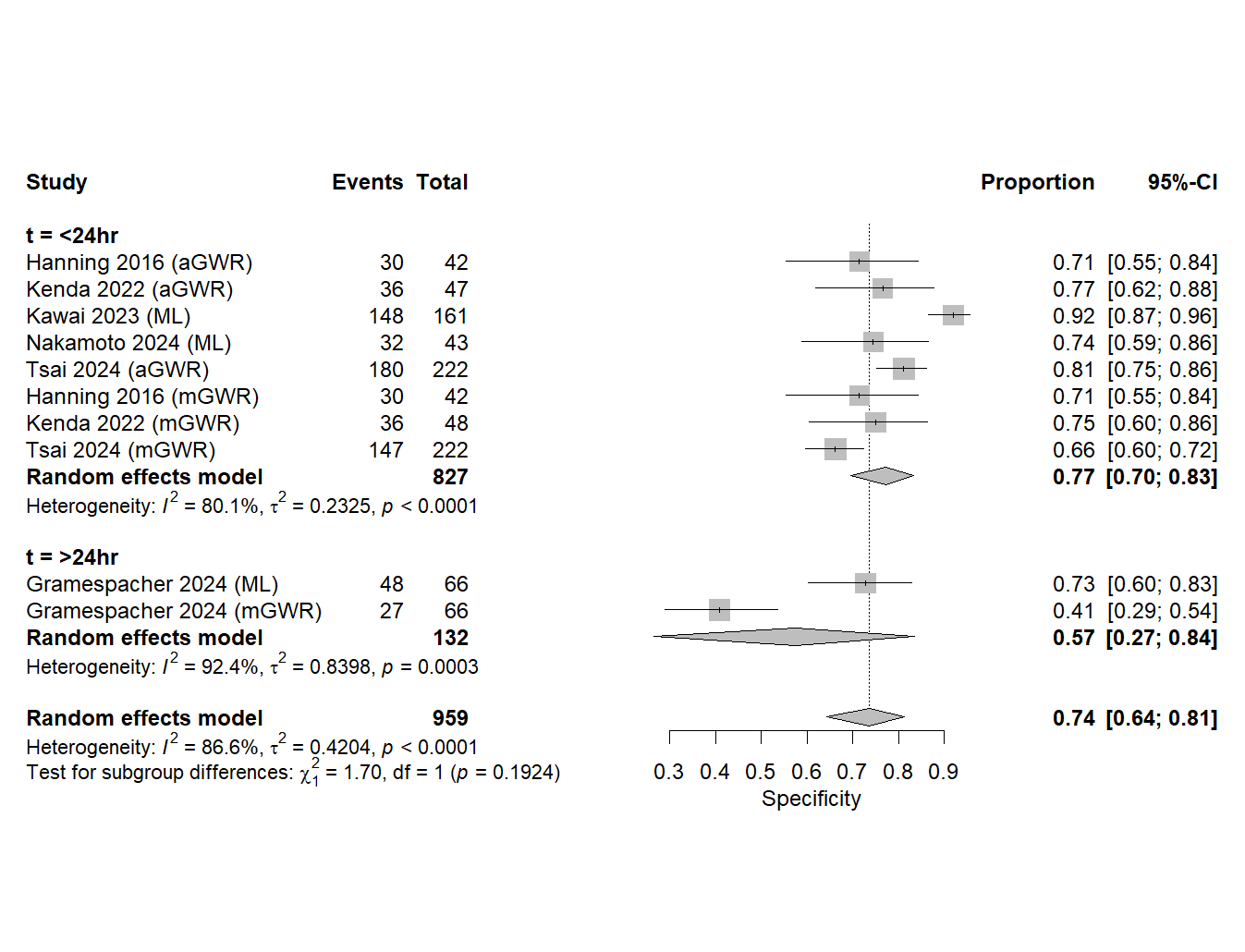

Supplement: Supplementary file 8 — Supplementary Material 8: Supplementary Fig. 5. Forest plot of the pooled specificity of brain computed tomography timing before and after the 24-h mark. Subgroup differences were not significant (p = 0.1924). [file 13049_2026_1565_MOESM8_ESM.tiff]

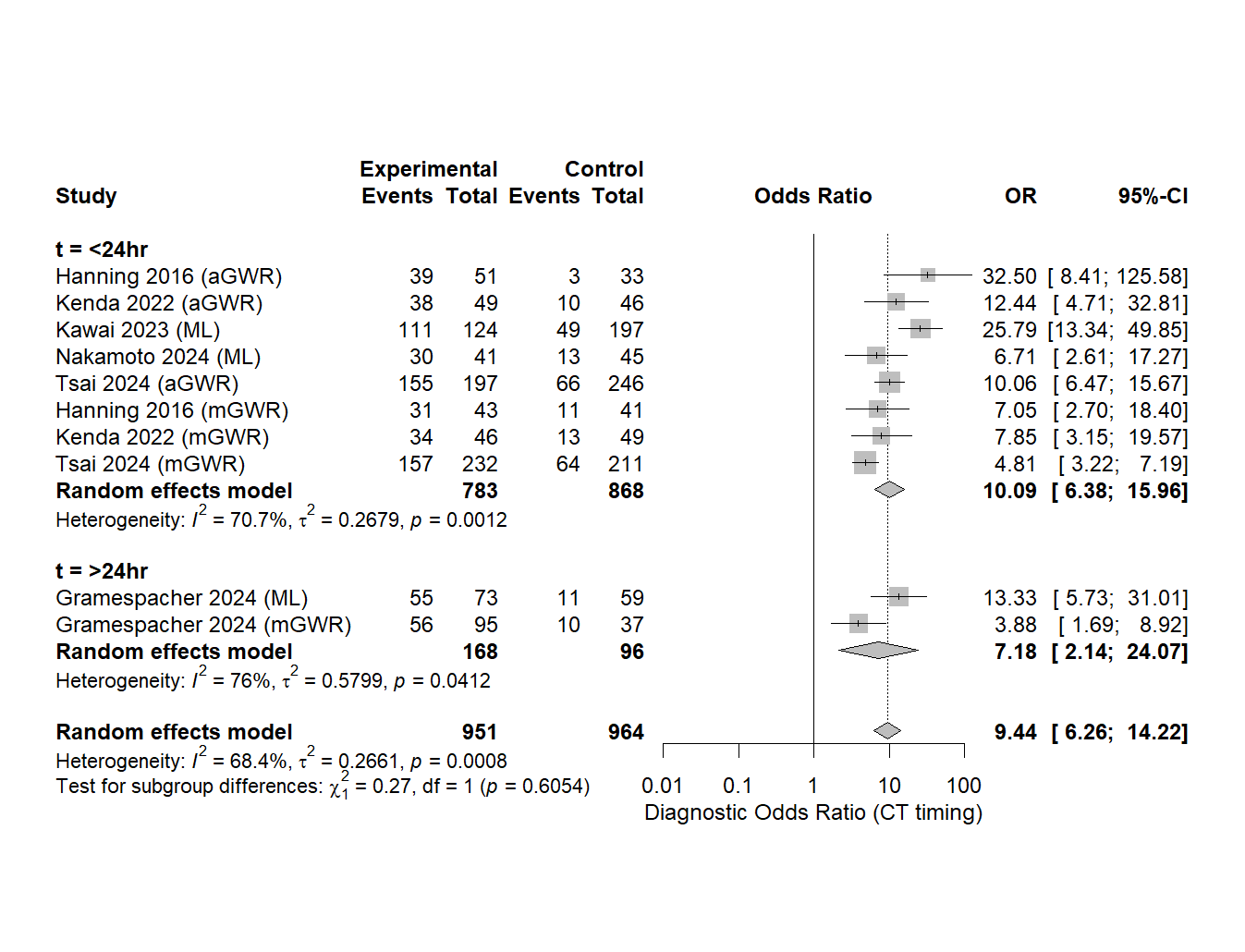

Supplement: Supplementary file 9 — Supplementary Material 9: Supplementary Fig. 6. Forest plot of the pooled diagnostic odds ratio of brain computed tomography timing before and after the 24-h mark. Subgroup differences were not significant (p = 0.6054). Abbreviations: ML, machine learning aGWR, automatic measurement of gray-to-white matter ratio; mGWR, manual measurement of gray-to-white matter ratio; CT, computed tomography; AUC, area under the curve. # Test before and after the 24-h mark in the ML group: p = 0.9689. # Test before and after the 24-h mark in the aGWR: Not available. # Test before and after the 24-h mark in the mGWR: p = 0.4681. # CT timing < 24 h: pooled AUC 0.734, I2 = 0%. # CT timing > 24 h: pooled AUC 0.835, I2 = 0%. [file 13049_2026_1565_MOESM9_ESM.tiff]
